# Can blood-based markers predict RECIST progression in non-small cell lung cancer treated with immunotherapy?

**DOI:** 10.1007/s00432-024-05814-2

**Published:** 2024-06-26

**Authors:** Melda Yeghaian, Teresa M. Tareco Bucho, Melissa de Bruin, Alexander Schmitz, Zuhir Bodalal, Egbert F. Smit, Regina G. H. Beets-Tan, Daan van den Broek, Stefano Trebeschi

**Affiliations:** 1 GROW Research Institute for Oncology and Reproduction, Maastricht University, Maastricht, The Netherlands; 2 Radiology Department, Netherlands Cancer Institute, Amsterdam, The Netherlands; 3 Pulmonology Department, Leiden University Medical Center, Leiden, The Netherlands; 4 Department of Laboratory Medicine, Netherlands Cancer Institute, Amsterdam, The Netherlands; 5 Faculty of Health Sciences, University of Southern Denmark, Odense, Denmark

**Keywords:** Blood-based markers, Progression-free survival, RECIST, NSCLC, Machine learning, Immunotherapy

## Abstract

**Purpose:**

In this study, we aimed to evaluate the potential of routine blood markers, serum tumour markers and their combination in predicting RECIST-defined progression in patients with stage IV non-small cell lung cancer (NSCLC) undergoing treatment with immune checkpoint inhibitors.

**Methods:**

We employed time-varying statistical models and machine learning classifiers in a Monte Carlo cross-validation approach to investigate the association between RECIST-defined progression and blood markers, serum tumour markers and their combination, in a retrospective cohort of 164 patients with NSCLC.

**Results:**

The performance of the routine blood markers in the prediction of progression free survival was moderate. Serum tumour markers and their combination with routine blood markers generally improved performance compared to routine blood markers alone. Elevated levels of C-reactive protein (CRP) and alkaline phosphatase (ALP) ranked as the top predictive routine blood markers, and CYFRA 21.1 was consistently among the most predictive serum tumour markers. Using these classifiers to predict overall survival yielded moderate to high performance, even when cases of death-defined progression were excluded. Performance varied across the treatment journey.

**Conclusion:**

Routine blood tests, especially when combined with serum tumour markers, show moderate predictive value  of RECIST-defined progression in NSCLC patients receiving immune checkpoint inhibitors. The relationship between overall survival and RECIST-defined progression may be influenced by confounding factors.

**Supplementary Information:**

The online version contains supplementary material available at 10.1007/s00432-024-05814-2.

## Introduction

Clinical trials are necessary to evaluate the efficacy and safety of new drugs in the treatment of non-small cell lung cancer (NSCLC). Overall survival (OS) is a desirable endpoint for assessing the effectiveness of cancer therapy (Driscoll and Rixe 2009). However, OS requires a lengthy follow-up, and death can occur due to various causes unrelated to cancer treatment. As an alternative, endpoints based on tumour size changes, such as progression-free survival (PFS), can be measured at earlier time points and have often replaced OS as an endpoint in clinical trials (Mushti et al. 2018). An accurate definition and assessment of disease progression are, therefore, vital in evaluating treatment efficacy in NSCLC patients. The Response Evaluation Criteria in Solid Tumours (RECIST) (Eisenhauer et al. 2009) is a commonly used framework to define progression, defined as either an increase in the size of target lesions (above 20%), an unequivocal increase of non-target lesions’ size, appearance of new lesions, or death. In immunotherapy, progression must be reassessed and confirmed in the subsequent follow-up (Seymour et al. 2017).

Routine blood tests are not invasive and are easily accessible as part of the routine monitoring of cancer patients during treatment (Bilen et al. 2019; Farina et al. 2023). Given that these are an already established tool in standard cancer patient management, leveraging them for the prediction of progression according to RECIST could offer a potentially cheaper and faster method to identify progression, compared to imaging assessments.

In NSCLC, research on the association between routine blood markers and progression has focused on inflammatory markers (Tanizaki et al. 2018; Iivanainen et al. 2019; Inomata et al. 2020; Peng et al. 2020; Chen et al. 2023), including the C reactive protein (CRP) and the neutrophil/lymphocyte ratio, lactate dehydrogenase (LDH) (Tanizaki et al. 2018; Inomata et al. 2020; Zhang et al. 2022) and albumin (Li et al. 2022; Menekse et al. 2023). Zhou et al. (2022) found good performance in the prediction of fast progression (significant progression occurring soon after the initiation of treatment) by a support vector machine model trained on laboratory test values, specifically on a four biomarker panel composed of neutrophil, CRP, LDH and alanine aminotransaminase (ALT). Fast progression patients predicted by this model were also found to have poor OS and PFS.

Serum tumour markers, produced by or in response to the tumour, have also been studied for monitoring both response and non-response, as well as for assessing the outcome of early treatment in immunotherapy-treated advanced-stage NSCLC (Essink et al. 2016; Lang et al. 2019; Dall’Olio et al. 2020; Muller et al. 2021; van Delft et al. 2022).

The objective of this study is to evaluate the potential of routine blood values in the prediction of RECIST progression in a longitudinal cohort of NSCLC patients receiving immune checkpoint inhibitors and to determine if combining routine with serum tumour markers offers additional advantages.

## Materials and methods

### Study cohort

We retrospectively collected stage-IV NSCLC patients treated with checkpoint inhibitors at the Netherlands Cancer Institute-Antoni van Leeuwenhoek Hospital (NKI-AVL; Amsterdam, The Netherlands) between 2014 and 2016, as a multi-modal (i.e. radiological + blood values) expansion of the dataset presented by Trebeschi et al. (2021). Patient and treatment characteristics are reported in Table 1. We retrieved blood data acquired for these patients between 2013 and 2018, collecting all routinely available measurements of blood markers from routine blood tests and serum tumour markers measured in the blood. A complete list of the markers collected can be accessed in the supplements. Progression-free survival was calculated from start of treatment to the date of RECIST 1.1 progression or death.

**Table 1:** Cohort characteristics

*Age*	
Median (years) (min, max)	64 (37, 83)
*Sex*	
Male	96 (58.5%)
Female	68 (41.5%)
*Tumor location diagnosis*	
Upper lobe, bronchus or lung	67
Lower lobe, bronchus or lung	33
Bronchus or lung, unspecified	10
Middle lobe, bronchus or lung	4
Main bronchus	4
Multiple locations	10
Unknown	36
*Treatment*	
Nivolumab	164
*Outcomes*	
Progression	139 (85%)
Death	128 (78%)
Median time to progression (days)	118
Median time to death (days)	303

### Time-varying regression analysis

In order to investigate whether blood markers were linked to RECIST progression during treatment, we used a statistical model named time-varying Cox regression (t-vCR) (Zhang et al. 2018). This model considers the entire time period between the start of treatment and the date when progression occurred, and analyses fluctuations in blood values during that time period to determine if they are significantly related to progression-free survival. The hazard ratio of each covariate (each blood value) will reflect the overall effect on the hazard rate considering that the covariate’s value may vary over time. Non-numerical blood values were filtered out or replaced by lower or upper bound values whenever applicable, as detailed in the supplements. Blood tests containing at least 25% of the available markers were used. We addressed remaining missing data using an unsupervised, iterative imputer based on Bayesian Ridge regression (Buck 1960; van Buuren and Groothuis-Oudshoorn 2011). To ensure convergence of the t-vCR model, we normalised the variables to a 0–1 range using the MinMaxScaler from Scikit-learn (Pedregosa et al. 2011).

### Machine learning pipeline

**Fig. 1:**
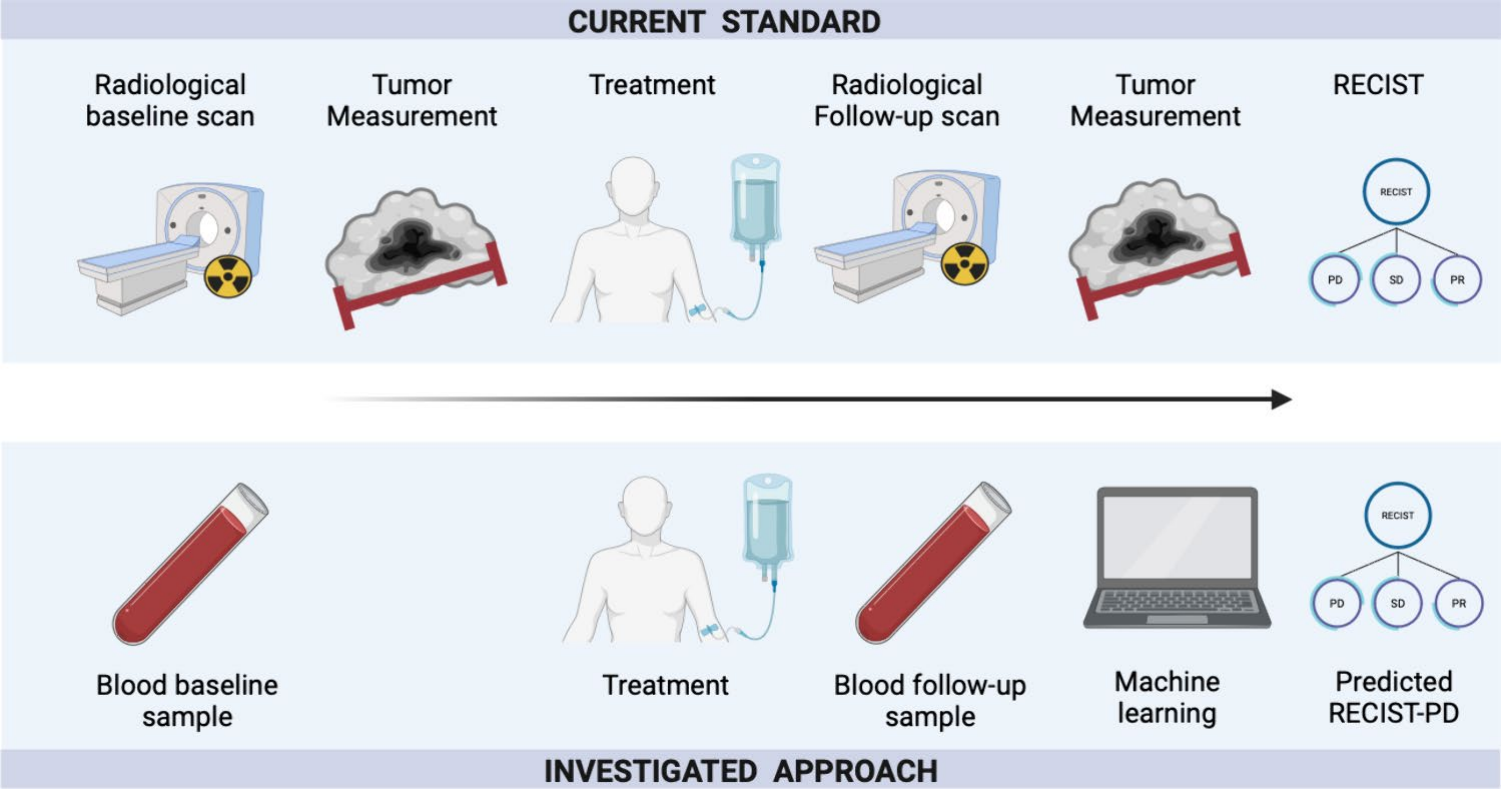
Scheme of the study design

To study if these markers could predict progression, we employed several classifiers, including random forests (RF) (Breiman 2001), and we trained them to predict whether progression occurs concurrently or will occur within different future time points: the next month, the next three, six, nine and twelve months from the date of the individual blood test. Each endpoint was trained and validated independently. Hyperparameter tuning was conducted for a subset of random forest parameters in all cross-validation splits, as detailed in the supplements. To predict concurrent progression (at the time of blood test acquisition), we incorporated all blood tests conducted from the start of treatment until three months after the recorded RECIST progression. For predicting progression at future time points, we included all blood tests conducted between three months prior to the start of treatment and up to three months after (or until the recorded progression, whichever happened first). Missing data was addressed using the same approach as detailed in the preceding section. Shapely Additive exPlanation (SHAP) values were used to explain the decisions of the RF classifier based on the validation sets (Lundberg et al. 2020). We used Python 3.9.7 for our analysis. Scikit-learn 1.0.2 (Pedregosa et al. 2011), Lifelines 0.27.4 (Creators Davidson-Pilon 2022), NumPy 1.21.6, Pandas 1.3.5 and SHAP 0.41.0 (Lundberg et al. 2020) were used for the implementation of the methods. To assess the robustness of the findings, we also repeated the analysis replacing the RF with support vector machines (SVM) (Hearst et al. 1998), and logistic regression. The study design is shown in Fig. 1.

### Validation strategy

Patient timelines can differ significantly, and traditional methods such as train/test splits or K-fold cross-validation on smaller sets may produce biased results depending on the specific split used. To avoid such biases, we utilised Monte Carlo cross-validation (MCCV) to estimate model performance (Xu and Liang 2001). With MCCV, we can randomly and reproducibly divide the dataset into multiple training (80%) and validation (20%) sets a certain number of times until stable and consistent performance estimates are obtained. This approach helps to ensure a reliable and unbiased evaluation of the model's performance. In our case, splitting is done on a patient-level on the raw data, after imputation of missing values. Results are reported in median performance (area under the curve (AUC), sensitivity, specificity, positive predictive value (PPV) and negative predictive value (NPV), at the median classification probability) across 100 MCCV folds with 95% confidence intervals. The code is shared publicly.^1^

## Results

Routine blood markers were available for n = 164 patients. The median age of the patients was 64 years, and 58.5% were male. Radiological progression was recorded in n = 139 patients. Serum tumour markers were available for n = 70 patients (43%), showing similar demographics: a median age of 64 years and 63% being male. The dataset for routine blood markers consisted of 3923 longitudinal routine blood tests, with a median of 21 blood tests (each blood test containing the measurements of multiple markers) collected from each patient at a median interval of approximately 14 days. A complete list of the utilised 31 routine blood markers and their abbreviations are reported in Supplementary Table 1. The subset of tumour markers dataset consisted of 1509 longitudinal blood tests, with each patient receiving a median of 20 tests at approximately 25-day median intervals. A total of five serum tumour markers were included: cytokeratin 19 fragment antigen (CYFRA 21.1), carcinoembryonic antigen (CEA), neuron-specific enolase (NSE), cancer antigen 125 (CA125), and squamous cell carcinoma antigen (SCC), with the first three being the most prevalent in our dataset. Validation details for each endpoint in each MCCV fold are reported in the supplements.

### Association between blood markers and PFS

Time-varying regression analysis was employed to study longitudinal links between blood markers and progression (Supplementary Tables 2 and 3). In routine blood values, increases in the levels of C-reactive protein (CRP) and alkaline phosphatase (ALP) were significantly associated with progression events (HR = 3.68, and 7.18; *p* = 0.018, and 0.018 respectively; Table 2). When combined with tumour markers, these blood values kept the same positive trends, but only CRP maintained its statistical significance (*p* = 0.011). Furthermore, CYFRA 21.1 also showed statistical significance (HR = 22.26, *p* = 0.008). The hazard ratios of the variables significantly associated with PFS are described in Table 2.

**Table 2:** Results of the time-varying Cox regression analysis

PFS		
Marker	HR (95% CI)	*p*-value
*Routine blood markers*		
CRP	3.68 (1.25–10.83)	0.018
ALP	7.18 (1.39–37.03)	0.018
*Routine and tumour markers*		
CRP	6.64 (1.54–28.63)	0.011
CYFRA 21.1	22.26 (2.28–217.41)	0.008

Only variables with significant effects for PFS in routine blood markers or in combination with tumour markers are shown and compared. HR = hazard ratio; CI = confidence interval. The effects of the remaining variables are provided in the supplementary tables 3 and 4

**Fig. 2:**
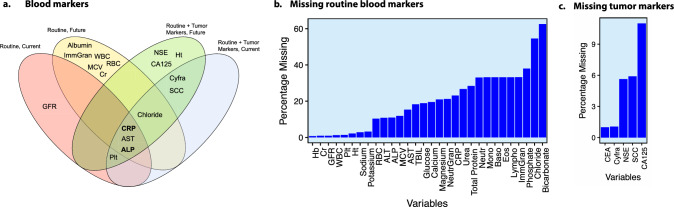
**a** Venn diagrams showing blood markers found related to RECIST progression across different analyses, namely the top predictive values for the SHAP analysis and the significant values for the time varying model analysis **b** Frequency of missing values in the dataset

To quantify the predictive value of blood markers to identify concurrent radiological progression, we used machine learning approaches. From now on, we refer to the results of the RF classifier. The results of SVMs and logistic regressions can be found in Supplementary Table 4, with comparable trends. Using only routine blood markers, we observed a moderate predictive value of 0.67 AUC (CI: 0.60–0.74). A similar result was observed when employing tumour markers, with an AUC of 0.67 (CI: 0.54–0.79). Combining routine blood and tumour markers increased the AUC to 0.69 (CI: 0.55–0.82). The AUC-based performance of the RF machine learning models is detailed in Table 3. Supporting metrics are provided in Supplementary Table 6. Using SHAP to uncover the most important markers for the machine learning models based on RFs (see Fig. 2a and Table 4), we observed ALP and CRP to be among the top most predictive markers for both models based on routine blood markers alone and on combined routine blood and tumour markers, and CYFRA 21.1 to follow in importance in the combined routine blood and tumour markers, suggesting an association of these markers with progression. SHAP summary plots for all prediction endpoints are included in the supplements (Supplementary Figs. 1–12).

**Table 3:** PFS and OS prediction performance using routine blood markers with/out combining tumour markers, with/out including death-defined progression cases

Data	Time point	AUC(95% CI)			
		PFS	OS	PFS (excluding death as PD)	OS (excluding death as PD)
Routine markers	Concurrent	0.67 (0.60–0.74)	–	–	–
	1-month	0.74 (0.65–0.82)	**0.85 (0.75**–**0.93)**	0.71 (0.61–0.81)	**0.84 (0.73**–**0.93)**
	3-month	**0.75 (0.69**–**0.81)**	0.79 (0.72–0.85)	0.73 (0.66–0.8)	0.79 (0.68–0.87)
	6-month	0.70 (0.64–0.77)	0.70 (0.64–0.77)	0.71 (0.64–0.78)	0.71 (0.63–0.78)
	9-month	0.71 (0.65–0.78)	0.70 (0.63–0.76)	**0.76 (0.68**–**0.82)**	0.74 (0.67–0.81)
	12-month	0.69 (0.62–0.76)	0.71 (0.65–0.77)	0.74 (0.66–0.81)	0.76 (0.69–0.82)
Tumour markers	Concurrent	0.67 (0.54–0.79)	–	–	–
	1-month	0.74 (0.54–0.9)	0.69 (0.52–0.85)	0.72 (0.53–0.88)	0.66 (0.54–0.78)
	3-month	**0.79 (0.67**–**0.9)**	**0.82 (0.68**–**0.93)**	**0.77 (0.63**–**0.89)**	**0.82 (0.67**–**0.94)**
	6-month	0.77 (0.66–0.87)	0.77 (0.66–0.87)	0.76 (0.64–0.86)	0.74 (0.61–0.85)
	9-month	0.78 (0.68–0.87)	0.74 (0.63–0.85)	**0.77 (0.64**–**0.87)**	0.73 (0.6–0.84)
	12-month	0.71 (0.59–0.82)	0.74 (0.62–0.84)	0.75 (0.63–0.86)	0.75 (0.62–0.86)
Routine + tumour markers	Concurrent	0.69 (0.55–0.82)	–	–	–
	1-month	0.83 (0.67–0.95)	0.87 (0.75–0.96)	**0.83 (0.67**–**0.95)**	**0.95 (0.9**–**0.98)**
	3-month	**0.86 (0.74**–**0.95)**	**0.89 (0.77**–**0.97)**	0.81 (0.66–0.93)	0.88 (0.76–0.96)
	6-month	0.83 (0.71–0.92)	0.78 (0.65–0.89)	0.80 (0.66–0.91)	0.74 (0.58–0.88)
	9-month	0.79 (0.66–0.89)	0.76 (0.63–0.87)	0.78 (0.63–0.90)	0.74 (0.59–0.87)
	12-month	0.72 (0.57–0.85)	0.76 (0.63–0.87)	0.73 (0.57–0.87)	0.77 (0.62–0.89)

**Table 4:** Most predictive blood markers (in order of importance) for RECIST-based PFS prediction according to SHAP using routine blood data alone and combined with serum tumour markers

Data	Time point	Predictive marker 1	Predictive marker 2	Predictive marker 3	Predictive marker 4	Predictive marker 5
*Predicting progression-free survival*						
Routine blood markers	Current	AST	ALP	GFR	CRP	Plt
	1-month	ALP	Albumin	ImmGran	MCV	AST
	3-month	ALP	CRP	AST	Chloride	WBC
	6-month	ALP	Plt	Chloride	CRP	AST
	9-month	ALP	CRP	Plt	Cr	AST
	12-month	ALP	CRP	AST	Chloride	RBC
Routine and tumour markers	Current	ALP	CYFRA 21.1	Chloride	CRP	SCC
	1-month	ALP	CYFRA 21.1	SCC	CA125	NSE
	3-month	CYFRA 21.1	CA125	Plt	ALP	Ht
	6-month	CYFRA 21.1	CA125	Plt	CRP	Ht
	9-month	CA125	CYFRA 21.1	SCC	CRP	AST
	12-month	ALP	CYFRA 21.1	Chloride	CRP	SCC
*Excluding death as progression*						
Routine blood markers	1-month	ALP	Albumin	CRP	MCV	AST
	3-month	ALP	CRP	Chloride	AST	Plt
	6-month	ALP	Chloride	Plt	AST	CRP
	9-month	ALP	Cr	GFR	CRP	Plt
	12-month	ALP	RBC	CRP	AST	Chloride
Routine and tumour markers	1-month	ALP	CRP	Magnesium	WBC	CYFRA 21.1
	3-month	ALP	CYFRA 21.1	SCC	CA125	CRP
	6-month	CYFRA 21.1	CA125	Plt	ALP	Ht
	9-month	CYFRA 21.1	CA125	Plt	Ht	Magnesium
	12-month	CA125	CYFRA 21.1	SCC	ALP	AST

### Relation between predictive blood markers and PFS

**Fig. 3:**
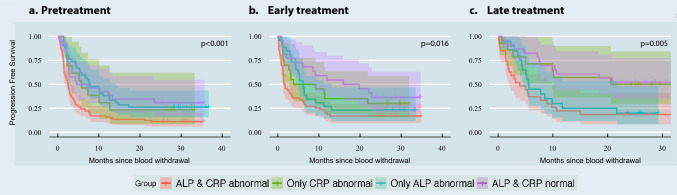
Survival KM curves of normal/abnormal CRP and ALP groups, as measured at **a** before start of treatment, **b** early treatment and **c** late during the treatment

Given the frequency of ALP and CRP emerging as important markers across the results of different types of RF analysis, we investigated further their relationship with PFS. We stratified the population of lab exams into subgroups based on the cutoff values used in our clinical workflow to define normal/abnormal ALP and CRP values: i.e. ALP > = 98 U/l for females, 115 U/l for males, and CRP > = 8 mg/l. This split created four subgroups: (1) normal-ALP normal-CRP, (2) normal-CRP abnormal-ALP, (3) abnormal-CRP normal-ALP, and (4) abnormal-ALP normal-CRP. Moreover, we split the analysis into the different stages of the treatment timeline, looking separately at the relationship between PFS and blood values collected pre-treatment (maximum one month), early on during the treatment (between 4 and 6 weeks), and at a later stage during treatment (between 10 and 12 weeks). Figure 3 shows the survival curves.

The group with abnormal CRP and ALP values showed shorter progression time, compared to the other subgroups, ranging from a median of 52 days for blood samples collected early during treatment to 123 days for blood samples collected later on during treatment (Supplementary Table 7). Interestingly, the normal CRP and ALP group showed lower median PFS pre-treatment (143 days; Fig. 3) than the groups with at most one abnormal value. During treatment, however, normalcy in both values was characteristic of longer PFS, reaching a median of 493 days, compared to 286 days of abnormal ALP group, 190 days of abnormal CRP group, and 52 days of abnormal CRP and ALP. Overall, no clear pattern of behaviour is visible among these groups, suggesting a complex relationship between CRP, ALP, and progression during treatment.

### Predicting future progression

**Fig. 4:**
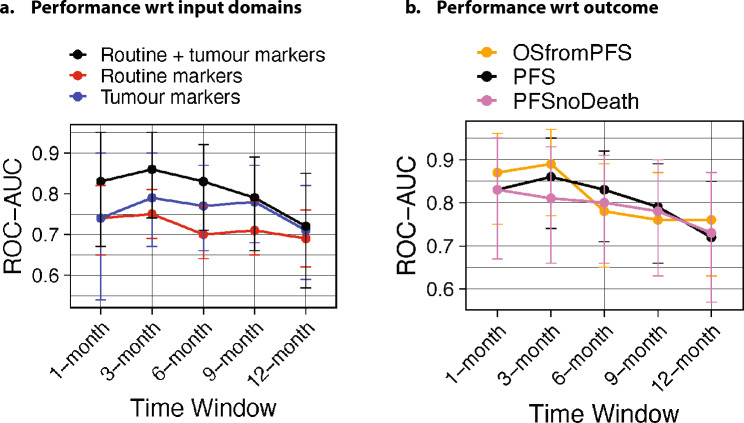
Comparative analysis of the performance with respect to **a** its inputs, and **b** its outputs

To investigate whether blood and tumour markers can *predict* future progression, we set up the machine learning pipeline to predict whether progression will happen within a given time frame from the moment of the blood sampling. The timeframes investigated were one, three, six, nine and twelve months. To predict progression within one month from the moment of blood withdrawal, the model reached an AUC of 0.74 for both routine markers, as well as tumour markers. The combination of routine markers and tumour markers yielded a higher performance, with an AUC of 0.83. To predict progression within three months from the moment of blood withdrawal, the model reached the highest performance in all three combinations with 0.86 AUC, observed in the combination of routine and tumour markers, being the highest. The performance of the model is then observed to decrease from three months on: from 0.75 to 0.69 AUC to predict progression within the next 12 months in blood markers-only models, from 0.79 to 0.71 AUC in tumour markers-only models, and from 0.86 to 0.72 AUC for combined tumour and blood markers models. Results are displayed in Fig. 4 and Table 3. ALP and CRP were also among the most important markers for predicting future progression, as seen in Table 4. Additional evaluation metrics are provided in Supplementary Table 5. The numbers of progressive patients in all validation sets for all studied endpoints and data modalities is reported in the supplements.

### Relationship between PFS and OS

We also investigated the relationship between PFS and OS in our cohort. In order to do that, we quantified the performance of our models, trained to predict PFS (PFS models), in the prediction of OS: if PFS and OS were to be independent variables, the models would not show any predictive value for the OS endpoint. Surprisingly, the models were observed to perform, on average, 3–4% AUC higher in the prediction of OS within three months from the moment of blood withdrawal in all modalities, up to 11% better performance for routine blood markers only models within one month from blood withdrawal (Table 3). Similar performances between PFS and OS were observed for the rest of the timeline.

These findings suggest a significant overlap of progression and the occurrence of death. Because progression in PFS is defined as either RECIST progression or death of the patient, these were analysed separately. We analysed the performance of the PFS models to predict RECIST progression only, and removed from the analysis the patients whose progression was defined by death. The models were retrained to ensure that no influence of death events can be traced into the models. We observed an average drop of 2–3% AUC in predictive performance of the models, up to 5% AUC drop for combined tumour and blood markers models within three months from the blood withdrawal. Similar performances between PFS and OS were observed for the rest of the timeline. Figure 4b shows the change in performances over time.

## Discussion

In this study, we aimed to assess the feasibility of using routine blood tests and serum tumour markers as a tool for defining radiological progression-free survival. Routine blood tests are non-invasive, non-ionizing, quantitative measurements not subject to variable interpretation. They are widely and frequently available and easily accessible in hospital settings, making them a compelling option for rapid monitoring of early treatment effects and tracking disease progression. Although genetic analysis of blood samples, such as circulating tumour DNA (ctDNA), has demonstrated the potential to identify radiological progression prior to imaging (Bettegowda et al. 2014; Hellmann et al. 2020), these require technical standardisation (Brozos-Vázquez et al. 2021), specialised equipment and highly trained personnel (Li et al. 2019).

Across all analyses, our findings revealed that, particularly, C-reactive protein (CRP) and alkaline phosphatase (ALP) were associated with radiological progression. In literature, a high level of pre-treatment CRP has been found to be a predictor of worse PFS and OS in NSCLC patients undergoing immunotherapy (Riedl et al. 2020). Moreover, CRP dynamics during treatment have also been found to be informative: early-treatment flares in the levels of CRP predict response (Klümper et al. 2022) and early decreases are associated with reduced risk of progression (Riedl et al. 2020). Additionally, fast increases over time and overall elevated trajectories of CRP have also been found to be predictors of high risk of progression (Riedl et al. 2020). While research on ALP has demonstrated their potential as biomarkers across different tumour types, its role in NSCLC remains uncertain (Jiang et al. 2023), and literature concerning NSCLC patients receiving immunotherapy is limited. Yang et al. (2023) found that higher levels of pre-treatment ALP are associated with lower overall response rate, and shorter median progression-free survival (mPFS) in patients with NSCLC treated with immune checkpoint inhibitors and that ALP is an independent prognostic indicator of mPFS. Julian et al. (2022) found that high levels of ALP were a risk increasing prognostic factor in advanced NSCLC patients receiving anti-PD-1/PD-L1 immune checkpoint inhibitors. In all analyses conducted in this study, there was a noticeable improvement in the predictive performance when routine blood and tumour markers were combined, reaching its peak at three months, compared to their individual performances. This highlights the complementary nature of the various blood-based biomarkers and the potential added value of incorporating them together. In the study of van Delft et al. (2022), CYFRA 21.1 was identified as the most predictive tumour marker for radiological response prediction at 6 months after treatment. Our study aligns with this finding for the prediction of radiological progression using combined blood markers, where CYFRA 21.1 consistently appears among the top two predictive markers across all time points. Despite the evident value of tumour markers, we nonetheless observed that ALP was more predictive (by SHAP) than CYFRA 21.1 for short-term events but also at one-year events. This example also shows how integrative analyses can be insightful in comparing different biomarkers with each other with respect to treatment endpoints. Integrating routine blood and tumour marker analysis with advanced techniques such as mass cytometry (CyTOF), that offer a comprehensive analysis of cellular phenotypes and functions at the single-cell level could also be relevant. CyTOF has emerged recently for the identification of circulating immune cell features associated with response (Mueller et al. 2022), and survival and progression outcomes (Rochigneux et al. 2022) of NSCLC patients receiving immunotherapy.

In a subsequent analysis, we looked further into the relationship between PFS and overall survival (OS) endpoints. Interestingly, our results indicated that the models tailored for PFS were more predictive for OS outcomes than anticipated in certain prediction time points. Routine blood parameters showed higher predictive power with shorter prediction windows, suggesting they may reflect the patient's ongoing overall health status, and other underlying conditions or acute illnesses not exclusively related to cancer (Crosbie et al. 2013). Conversely, tumour markers had an opposite pattern, with increasing predictive performance for longer prediction windows, implying that they may be more indicative of the early stages of tumour progression and its trajectory.

Indeed, the relationship between PFS and OS is complex and varies across different cancer types and therapeutics. In the context of NSCLC and immunotherapy or targeted therapies, previous studies suggest, at most, moderate potential for PFS to be used as a surrogate of OS (Ye et al. 2020; Shameer et al. 2021; Hua et al. 2022), while strongly advocating for the need for patient-level analyses, where true surrogacy can be evaluated.

Unlike OS, PFS, as assessed by RECIST, is subject to variability due to the inconsistent application of the criteria. The variability sources can pertain to, among others, reader experience (Tovoli et al. 2018), identification of new lesions (Beaumont et al. 2018), unidimensional measurement errors (Yoon et al. 2016), selection of target lesions (Iannessi et al. 2021) or non-target disease assessment (Morse et al. 2019). While some of these can be minimised by, for example, having automated methods for lesion measurements (Fournier et al. 2021) and multiple experienced readers (Karmakar et al. 2019; Schmid et al. 2021), the reader-subjective nature of RECIST, limited to the selection of target lesions, may not be eliminated. Without the guarantee of an objective definition of progression, PFS may be misidentified, potentially introducing noise into our data.

Our study has some limitations. Firstly, this was a single-centre study with no external validation, implying that our findings might not be directly translatable or generalisable to external cohorts without further testing. To mitigate this, we report the average performance of our models across 100 cross-validation splits. Moreover, the specificity of our cohort to NSCLC and its treatment with immunotherapy may limit the applicability of our results to other types of cancers or treatments. Larger cohorts, with appropriate sample size estimations and additional study contexts, will be required to confirm our results.

## Conclusions

Our study highlights the potential of using routine blood tests as a tool for predicting progression free survival in NSCLC cancer patients receiving immune checkpoint inhibitors. Integrating routine blood markers and serum tumour markers within machine learning models may enable more performant and timely detection of disease progression, ultimately aiding clinical decision-making. Further studies with larger cohorts are needed to validate these findings and explore the generalizability of our approach to other cancer types and treatment modalities.

### Supplementary Information

Below is the link to the electronic supplementary material.

Supplementary file1 (DOCX 2452 KB)

Supplementary file2 (XLSX 77 KB)

Supplementary file3 (XLSX 303 KB)

## Data Availability

The data that support the findings of this study are available from the corresponding author, S.T., upon reasonable request.

## Footnotes

1 github.com/nki-radiology/BloodRecistProgression
