# Supplementary material for: Can blood-based markers predict RECIST progression in non-small cell lung cancer treated with immunotherapy?
Source: J Cancer Res Clin Oncol. 2024 Jun 26;150(6):329. doi: 10.1007/s00432-024-05814-2 (PMC11208229; doi:10.1007/s00432-024-05814-2)

# **Supplementary Materials:**

The total routine blood markers were:

Hemoglobin (Hb), hematocrit (Ht), mean corpuscular volume (MCV), erythrocytes (RBC), thrombocytes (Plt), leukocytes (WBC), lymphocytes (Lympho; absolute count, automatic, and differential count), monocytes (Mono; absolute count, automatic count, and differential count), eosinophils (Eos; absolute count, automatic count, and differential count), basophils (Baso; absolute count, automatic count, and differential count), neutrophil counts (Neutr), a combination of neutrophils, basophils (absolute count, automatic count, and differential count) and neutrophyl granulocytes (NeutrGran), immature granulocytes (ImmGran), C-reactive protein (CRP), total bilirubin (TBIL), alkaline phosphatase (ALP), aspartate aminotransferase (AST), alanine aminotransferase (ALT) , creatinine (Cr), glomerular filtration rate (GFR), sodium, potassium, chloride, bicarbonate, phosphate, calcium, magnesium, urea, glucose, total protein, albumin, mean corpuscular hemoglobin (MCH), mean corpuscular hemoglobin concentration (MCHC), blasts, metamyelocytes, myelocytes, normoblasts, erythrocyte sedimentation rate, lactate, Interleukin-6 (IL-6), direct bilirubin, reticulocytes (absolute and differential count), erytroblasts, plasma cells, band cells (absolute and percent count), granulocytes segmental nuclear (absolute and percentage count).

In addition, 7 tumor markers were obtained, namely: cytokeratin 19 fragment antigen (CYFRA 21.1), carcinoembryonic antigen (CEA), neuron-specific enolase (NSE), cancer antigen 125 (CA125), and squamous cell carcinoma antigen (SCC), chromogranin A (CgA-1), prostate-specific antigen (PSA).

Filtered out all rows that had non numeric values in the features (eg. <mg, <hem, <aanw, <aggr). Non-numerical blood values representing ranges were replaced by lower or upper bound values, replacing <1, <2, <3, <0.1, <0.3, <3, <5 with 0.


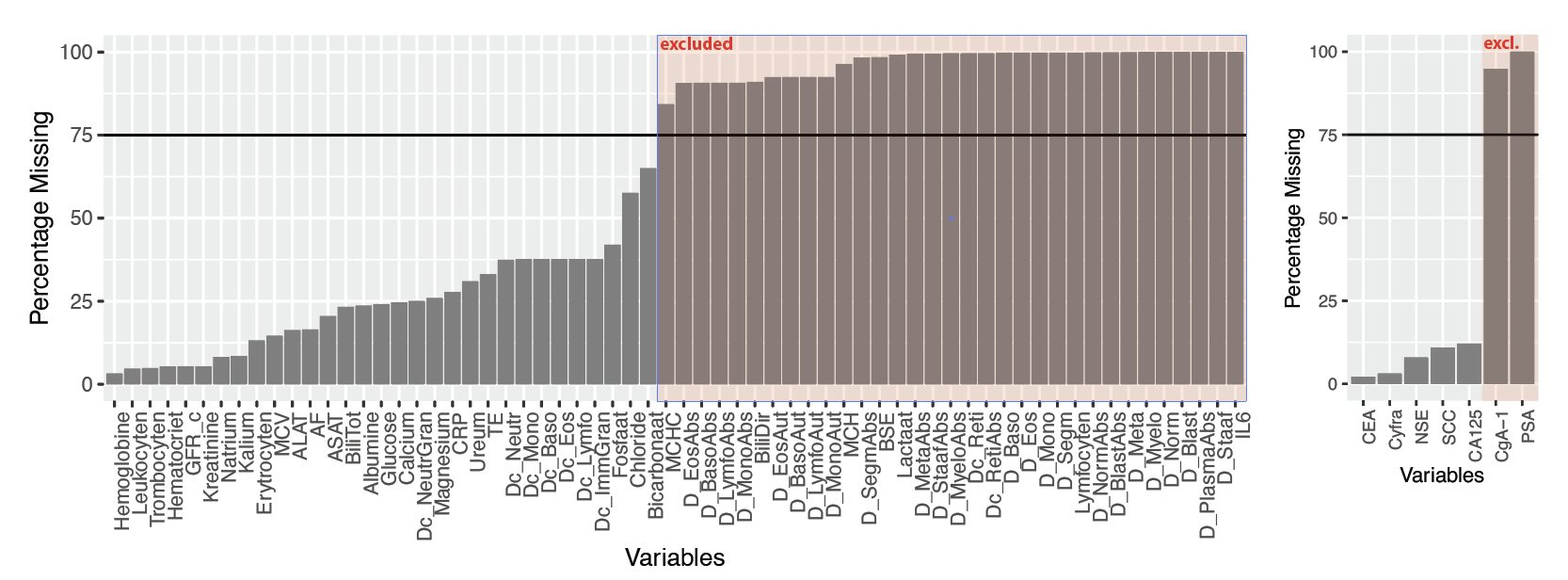


Table 1: A list of utilized routine blood markers and their abbreviations

| Blood marker | Abbreviation | Unit |
| --- | --- | --- |
| hemoglobin | Hb | mmol/l |
| hematocrit | Ht | l/l |
| mean corpuscular volume | MCV | (Ht/Rbc) fl |
| erythrocytes | RBC | 10E12/l |
| thrombocytes | Plt | 10E9/l |
| leukocytes | WBC | 10E9/l |
| lymphocytes | Lympho | % |
| monocytes | Mono | 10E9/l |
| eosinophils | Eos | % |
| basophils | Baso | % |
| neutrophil counts | Neutr | 10E9/l |
| a combination of neutrophils, basophils and eosinophils | NeutrGran | 10E9/l |
| immature granulocytes | ImmGran | 10E9/l |
| C-reactive protein | CRP | mg/l |
| total bilirubin | TBIL | µmol/l |
| alkaline phosphatase | ALP | U/l |
| aspartate aminotransferase | AST | U/l |
| alanine aminotransferase | ALT | U/l |
| creatinine | Cr | µmol/l |
| glomerular filtration rate | GFR | ml/min/1.73m2 |
| sodium | - | mmol/l |
| potassium | - | mmol/l |
| chloride | - | mmol/l |
| bicarbonate | - | mmol/l |
| phosphate | - | mmol/l |
| calcium | - | mmol/l |
| magnesium | - | mmol/l |
| urea | - | mmol/l |
| glucose | - | mmol/l |
| total protein | - | g/l |
| albumin | - | g/l |

Table 2: Results of the time-varying Cox regression analysis using routine blood markers. All variables are shown. HR = hazard ratio; CI = confidence interval. lab_test_from_SoT = number of days from start of treatment to the blood withdraw

| Covariate | HR | HR lower 95% | HR upper 95% | *p* |
| --- | --- | --- | --- | --- |
| lab_test_from_SoT | 1 | 1 | 1 | 0,854 |
| Hb | 0,72 | 0,37 | 1,38 | 0,318 |
| Ht | 0,7 | 0,35 | 1,39 | 0,307 |
| MCV | 0,83 | 0,38 | 1,78 | 0,626 |
| RBC | 0,77 | 0,41 | 1,44 | 0,407 |
| Plt | 1,5 | 0,59 | 3,86 | 0,396 |
| WBC | 2,51 | 0,5 | 12,71 | 0,265 |
| Lympho | 0,98 | 0,67 | 1,45 | 0,931 |
| Mono | 1,32 | 0,82 | 2,12 | 0,248 |
| Eos | 1,43 | 0,52 | 3,93 | 0,482 |
| Baso | 1,08 | 0,69 | 1,71 | 0,737 |
| Neutr | 1,3 | 0,7 | 2,42 | 0,412 |
| NeutrGran | 1,71 | 0,42 | 7,03 | 0,455 |
| ImmGran | 1,32 | 0,5 | 3,49 | 0,579 |
| CRP | 3,68 | 1,25 | 10,84 | 0,018 |
| TBIL | 3,05 | 0,09 | 108,7 | 0,541 |
| ALP | 7,18 | 1,39 | 37,03 | 0,018 |
| AST | 2,77 | 0,44 | 17,44 | 0,277 |
| ALT | 1,6 | 0,32 | 8,05 | 0,571 |
| Cr | 0,7 | 0,29 | 1,64 | 0,406 |
| GFR | 1,85 | 0,85 | 4 | 0,119 |
| Sodium | 0,78 | 0,34 | 1,77 | 0,547 |
| Potassium | 1,1 | 0,39 | 3,14 | 0,855 |
| Chloride | 0,5 | 0,2 | 1,21 | 0,124 |
| Bicarbonate | 1 | 0,33 | 3,02 | 0,998 |
| Phosphate | 0,73 | 0,2 | 2,68 | 0,636 |
| Calcium | 0,67 | 0,2 | 2,25 | 0,519 |
| Magnesium | 0,85 | 0,27 | 2,66 | 0,775 |
| Urea | 1,17 | 0,36 | 3,79 | 0,798 |
| Glucose | 1,71 | 0,37 | 7,86 | 0,488 |
| Total Protein | 0,92 | 0,39 | 2,17 | 0,843 |
| Albumin | 0,63 | 0,31 | 1,26 | 0,19 |

Table 3: Results of the time-varying Cox regression analysis using routine blood markers and tumour markers. All variables are shown. HR = hazard ratio; CI = confidence interval. lab_test_from_SoT = number of days from start of treatment to the blood withdraw

| Covariate | HR | HR lower 95% | HR upper 95% | *p* |
| --- | --- | --- | --- | --- |
| lab_test_from_SoT | 1 | 1 | 1 | 0,844 |
| Hb | 0,85 | 0,29 | 2,49 | 0,761 |
| Ht | 0,76 | 0,26 | 2,23 | 0,618 |
| MCV | 0,55 | 0,14 | 2,12 | 0,384 |
| RBC | 0,94 | 0,32 | 2,72 | 0,903 |
| Plt | 0,82 | 0,16 | 4,32 | 0,819 |
| WBC | 1,93 | 0,36 | 10,26 | 0,439 |
| Lympho | 1,34 | 0,42 | 4,25 | 0,62 |
| Mono | 1,1 | 0,26 | 4,67 | 0,895 |
| Eos | 0,74 | 0,03 | 15,95 | 0,845 |
| Baso | 0,9 | 0,32 | 2,54 | 0,846 |
| Neutr | 1,93 | 0,4 | 9,3 | 0,413 |
| NeutrGran | 1,93 | 0,4 | 9,29 | 0,414 |
| ImmGran | 0,8 | 0,18 | 3,63 | 0,772 |
| CRP | 6,64 | 1,54 | 28,63 | 0,011 |
| TBIL | 0,96 | 0 | 5123,48 | 0,992 |
| ALP | 10,63 | 0,19 | 609,37 | 0,253 |
| AST | 1,86 | 0,11 | 30,6 | 0,664 |
| ALT | 1,04 | 0,01 | 192,08 | 0,988 |
| Cr | 1,23 | 0,28 | 5,41 | 0,785 |
| GFR | 1,56 | 0,45 | 5,44 | 0,485 |
| Sodium | 0,6 | 0,17 | 2,1 | 0,422 |
| Potassium | 1,39 | 0,34 | 5,68 | 0,645 |
| Chloride | 0,47 | 0,1 | 2,24 | 0,341 |
| Bicarbonate | 0,5 | 0,09 | 2,64 | 0,414 |
| Phosphate | 0,87 | 0,2 | 3,87 | 0,858 |
| Calcium | 1,37 | 0,09 | 19,88 | 0,817 |
| Magnesium | 0,38 | 0,08 | 1,84 | 0,231 |
| Urea | 1,3 | 0,21 | 8,17 | 0,782 |
| Glucose | 2,28 | 0,51 | 10,11 | 0,28 |
| Total Protein | 1,13 | 0,28 | 4,62 | 0,861 |
| Albumin | 0,59 | 0,13 | 2,6 | 0,485 |
| CEA | 3,21 | 0,45 | 22,86 | 0,245 |
| Cyfra | 22,26 | 2,28 | 217,41 | 0,008 |
| NSE | 0,58 | 0,05 | 6,72 | 0,66 |
| SCC | 3,96 | 0,19 | 83,34 | 0,376 |
| CA125 | 4,08 | 0,87 | 19,18 | 0,075 |

Table 4: RECIST-based PFS prediction performance using different machine learning models

| Data | Time point | AUC(95% CI) | | |
| --- | --- | --- | --- | --- |
|  |  | Random forest | Support vector machine | Logistic regression |
| Routine markers | concurrent | **0.67 (0.60 - 0.74)** | 0.57 (0.49 - 0.66) | 0.60 (0.52 - 0.67) |
|  | 1-month | **0.74 (0.65 - 0.82)** | 0.71 (0.61 - 0.80) | **0.74 (0.66 - 0.82)** |
|  | 3-month | **0.75 (0.69 - 0.81)** | 0.72 (0.65 - 0.78) | 0.70 (0.63 - 0.76) |
|  | 6-month | 0.70 (0.64 - 0.77) | **0.71 (0.64 - 0.77)** | 0.69 (0.62 - 0.75) |
|  | 9-month | 0.71 (0.65 - 0.78) | **0.73 (0.66 - 0.79)** | 0.72 (0.65 - 0.78) |
|  | 12-month | **0.69 (0.62 - 0.76)** | 0.67 (0.59 - 0.74) | 0.68 (0.6 - 0.75) |
| Tumour markers | concurrent | **0.67 (0.54 - 0.79)** | 0.53 (0.37 - 0.69) | 0.62 (0.48 - 0.76) |
|  | 1-month | **0.74 (0.54 - 0.9)** | 0.58 (0.36 - 0.8) | 0.68 (0.51 - 0.84) |
|  | 3-month | **0.79 (0.67 - 0.9)** | 0.70 (0.55 - 0.83) | 0.72 (0.59 - 0.84) |
|  | 6-month | 0.77 (0.66 - 0.87) | **0.80 (0.69 - 0.88)** | 0.77 (0.66 - 0.86) |
|  | 9-month | 0.78 (0.68 - 0.87) | 0.79 (0.68 - 0.88) | **0.80 (0.70 - 0.89)** |
|  | 12-month | 0.71 (0.59 - 0.82) | 0.67 (0.54 - 0.79) | **0.77 (0.66 - 0.87)** |
| Routine + tumour markers | concurrent | **0.69 (0.55 - 0.82)** | 0.55 (0.37 - 0.72) | 0.60 (0.44 - 0.75) |
|  | 1-month | **0.83 (0.67 - 0.95)** | 0.77 (0.55 - 0.94) | 0.69 (0.42 - 0.91) |
|  | 3-month | **0.86 (0.74 - 0.95)** | 0.83 (0.70 - 0.93) | 0.80 (0.65 - 0.92) |
|  | 6-month | 0.83 (0.71 - 0.92) | **0.84 (0.72 - 0.93)** | 0.78 (0.66 - 0.88) |
|  | 9-month | 0.79 (0.66 - 0.89) | **0.82 (0.70 - 0.91)** | 0.79 (0.67 - 0.89) |
|  | 12-month | 0.72 (0.57 - 0.85) | 0.73 (0.59 - 0.85) | **0.76 (0.63 - 0.88)** |

Table 5: RECIST-based PFS prediction performance using random forest and different evaluation metrics

| Data | Time point | PFS | | | | |
| --- | --- | --- | --- | --- | --- | --- |
|  |  | AUC(95% CI) | Sensitivity(95% CI) | Specificity(95% CI) | PPV(95% CI) | NPV(95% CI) |
| Routine markers | concurrent | 0.67  (0.60 - 0.74) | 0.53  (0.49 - 0.58) | 0.72  (0.61 - 0.82) | 0.19  (0.16 - 0.22) | 0.93  (0.9 - 0.95) |
|  | 1-month | 0.74  (0.65 - 0.82) | 0.55  (0.49 - 0.62) | 0.79  (0.66 - 0.91) | 0.23  (0.19 - 0.26) | 0.94  (0.9 - 0.97) |
|  | 3-month | **0.75**  **(0.69 - 0.81)** | 0.66  (0.58 - 0.73) | 0.72  (0.63 - 0.80) | 0.59  (0.54 - 0.66) | 0.77  (0.71 - 0.82) |
|  | 6-month | 0.70  (0.64 - 0.77) | 0.68  (0.59 - 0.77) | 0.62  (0.54 - 0.69) | 0.75  (0.69 - 0.8) | 0.53  (0.48 - 0.59) |
|  | 9-month | 0.71  (0.65 - 0.78) | 0.74  (0.64 - 0.83) | 0.60  (0.52 - 0.67) | 0.84  (0.79 - 0.89) | 0.43  (0.38 - 0.49) |
|  | 12-month | 0.69  (0.62 - 0.76) | 0.72  (0.61 - 0.83) | 0.57  (0.5 - 0.64) | 0.86  (0.81 - 0.91) | 0.35  (0.3 - 0.4) |
| Tumour markers | concurrent | 0.67  (0.54 - 0.79) | 0.53  (0.44 - 0.61) | 0.69  (0.48 - 0.88) | 0.15  (0.1 - 0.19) | 0.93  (0.89 - 0.97) |
|  | 1-month | 0.74  (0.54 - 0.9) | 0.53  (0.42 - 0.64) | 0.82  (0.58 - 0.99) | 0.14  (0.09 - 0.19) | 0.97  (0.93 - 1.0) |
|  | 3-month | **0.79**  **(0.67 - 0.9)** | 0.62  (0.50 - 0.74) | 0.79  (0.62 - 0.93) | 0.45  (0.36 - 0.55) | 0.88  (0.80 - 0.96) |
|  | 6-month | 0.77  (0.66 - 0.87) | 0.70  (0.56 - 0.83) | 0.70  (0.56 - 0.83) | 0.68  (0.58 - 0.79) | 0.70  (0.60 - 0.80) |
|  | 9-month | 0.78  (0.68 - 0.87) | 0.75  (0.61 - 0.88) | 0.67  (0.54 - 0.80) | 0.78  (0.69 - 0.88) | 0.61  (0.52 - 0.72) |
|  | 12-month | 0.71  (0.59 - 0.82) | 0.74  (0.57 - 0.89) | 0.60  (0.47 - 0.73) | 0.83  (0.74 - 0.91) | 0.45  (0.36 - 0.55) |
| Routine + tumour markers | concurrent | 0.69  (0.55 - 0.82) | 0.53  (0.44 - 0.63) | 0.76  (0.53 - 0.95) | 0.16  (0.11 - 0.21) | 0.95  (0.91 - 0.99) |
|  | 1-month | 0.83  (0.67 - 0.95) | 0.55  (0.41 - 0.68) | 0.90  (0.75 - 1.00) | 0.17  (0.12 - 0.24) | 0.98  (0.95 - 1.0) |
|  | 3-month | **0.86**  **(0.74 - 0.95)** | 0.66  (0.52 - 0.80) | 0.85  (0.68 - 0.97) | 0.53  (0.42 - 0.65) | 0.90  (0.81 - 0.98) |
|  | 6-month | 0.83  (0.71 - 0.92) | 0.75  (0.59 - 0.89) | 0.74  (0.58 - 0.87) | 0.74  (0.63 - 0.86) | 0.72  (0.61 - 0.83) |
|  | 9-month | 0.79  (0.66 - 0.89) | 0.77  (0.6 - 0.91) | 0.67  (0.51 - 0.81) | 0.80  (0.70 - 0.91) | 0.59  (0.48 - 0.72) |
|  | 12-month | 0.72  (0.57 - 0.85) | 0.74  (0.54 - 0.92) | 0.59  (0.44 - 0.73) | 0.84  (0.74 - 0.93) | 0.43  (0.32 - 0.55) |

Table : Comparison of the median progression free survival and overall survival time (in days) between the different subgroups of tests with normal and abnormal CRP and ALP levels.

| Endpoint | Time point | Normal CRP Normal ALP | Normal CRP Abnormal ALP | Abnormal CRP  Normal ALP | Abnormal CRP  Abnormal ALP |
| --- | --- | --- | --- | --- | --- |
| PFS | Pre-treatment | 143 | 178 | 239 | 77 |
|  | Early on-treatment | 493 | 286 | 190 | 52 |
|  | Later on-treatment | - | - | 174 | 123 |
| OS | Pre-treatment | 524 | 441 | 436 | 155 |
|  | Early on-treatment | - | 577 | 337 | 230 |
|  | Later on-treatment | - | - | 483 | 251 |

(-) median not reached

Supplementary Figures:

Figure 1: SHAP summary plot of current progression prediction using routine blood markers


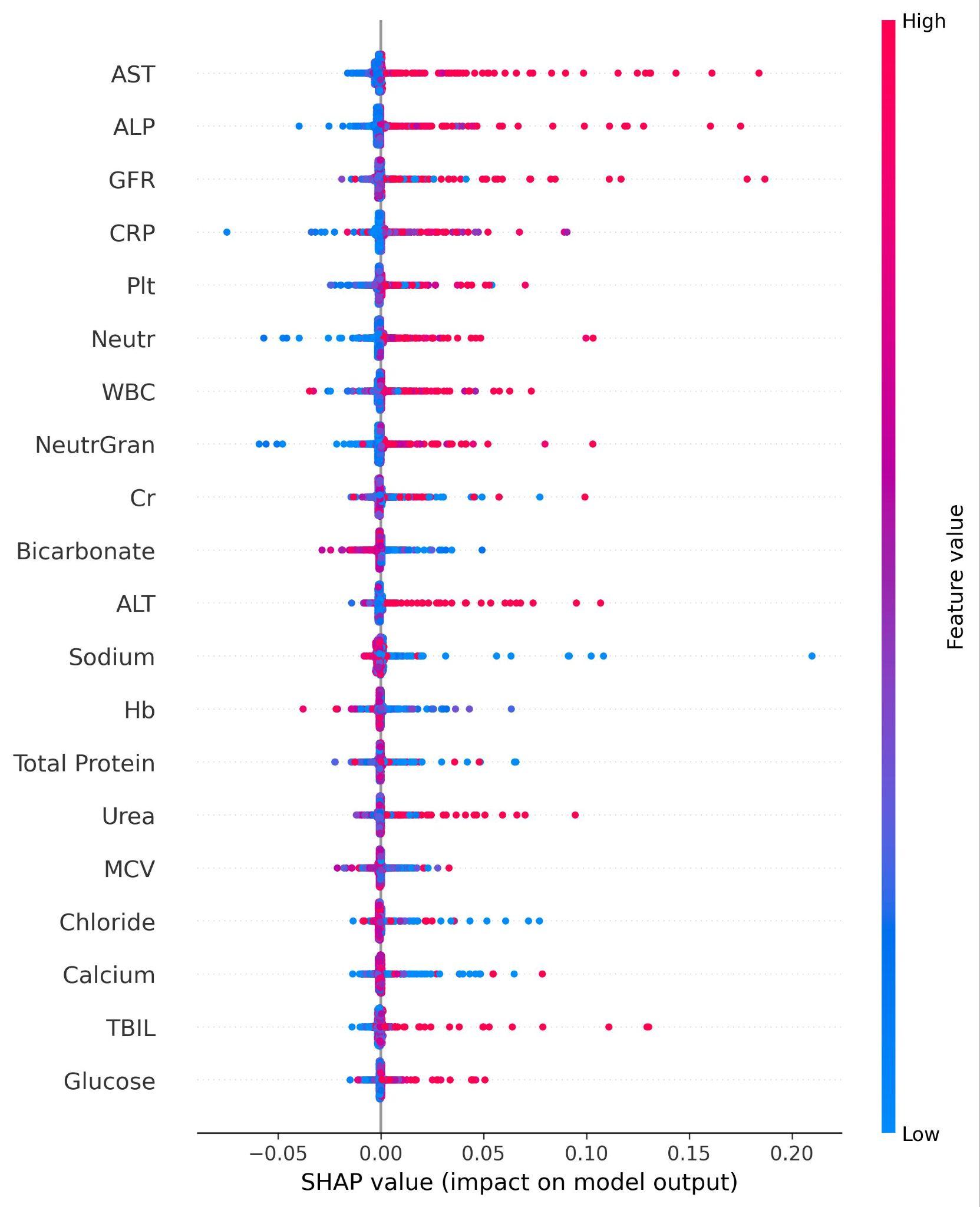


Figure 2: SHAP summary plot of current progression prediction using combined routine blood markers and tumour markers


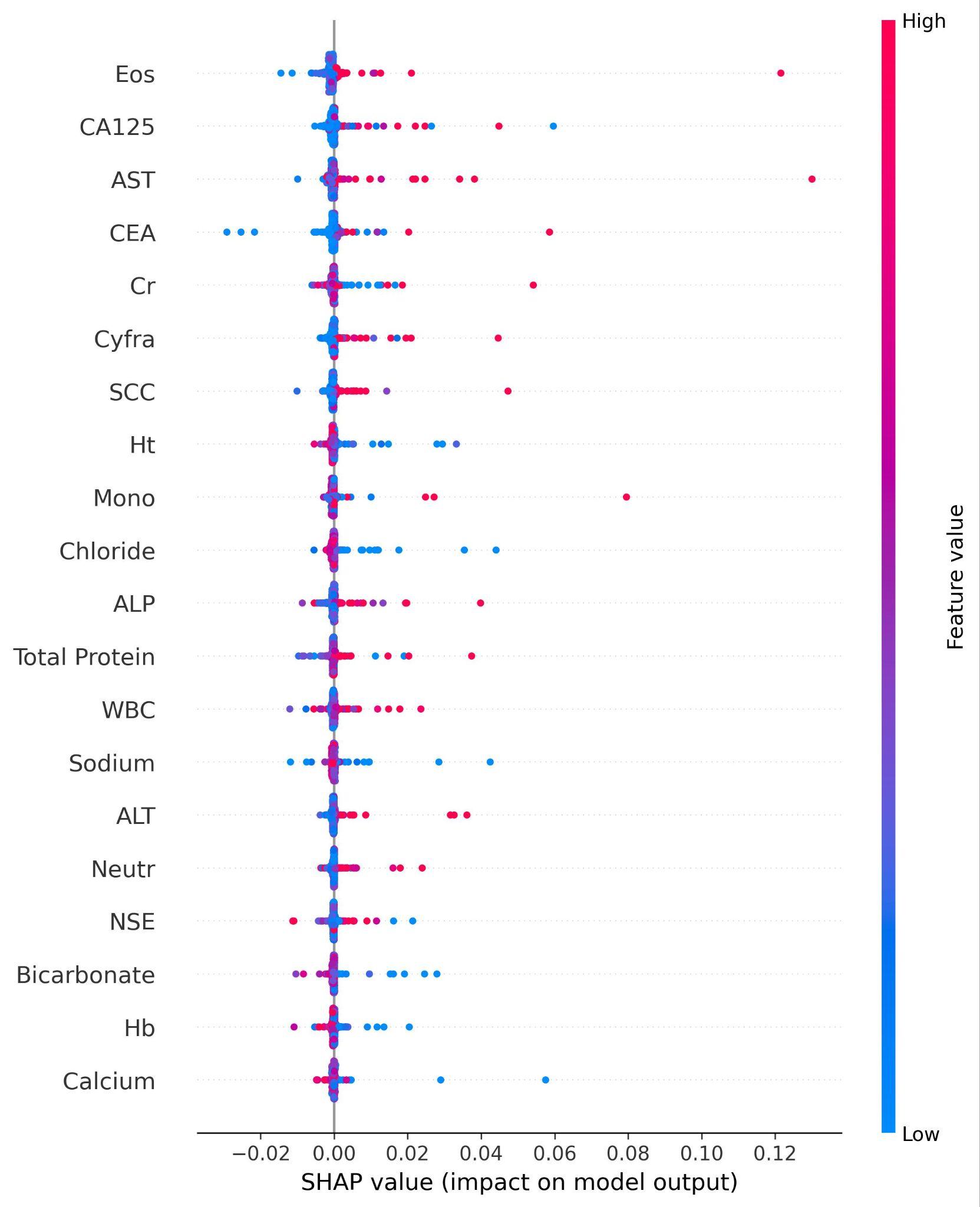


Figure 3: SHAP summary plot of 1-month pfs prediction using routine blood markers


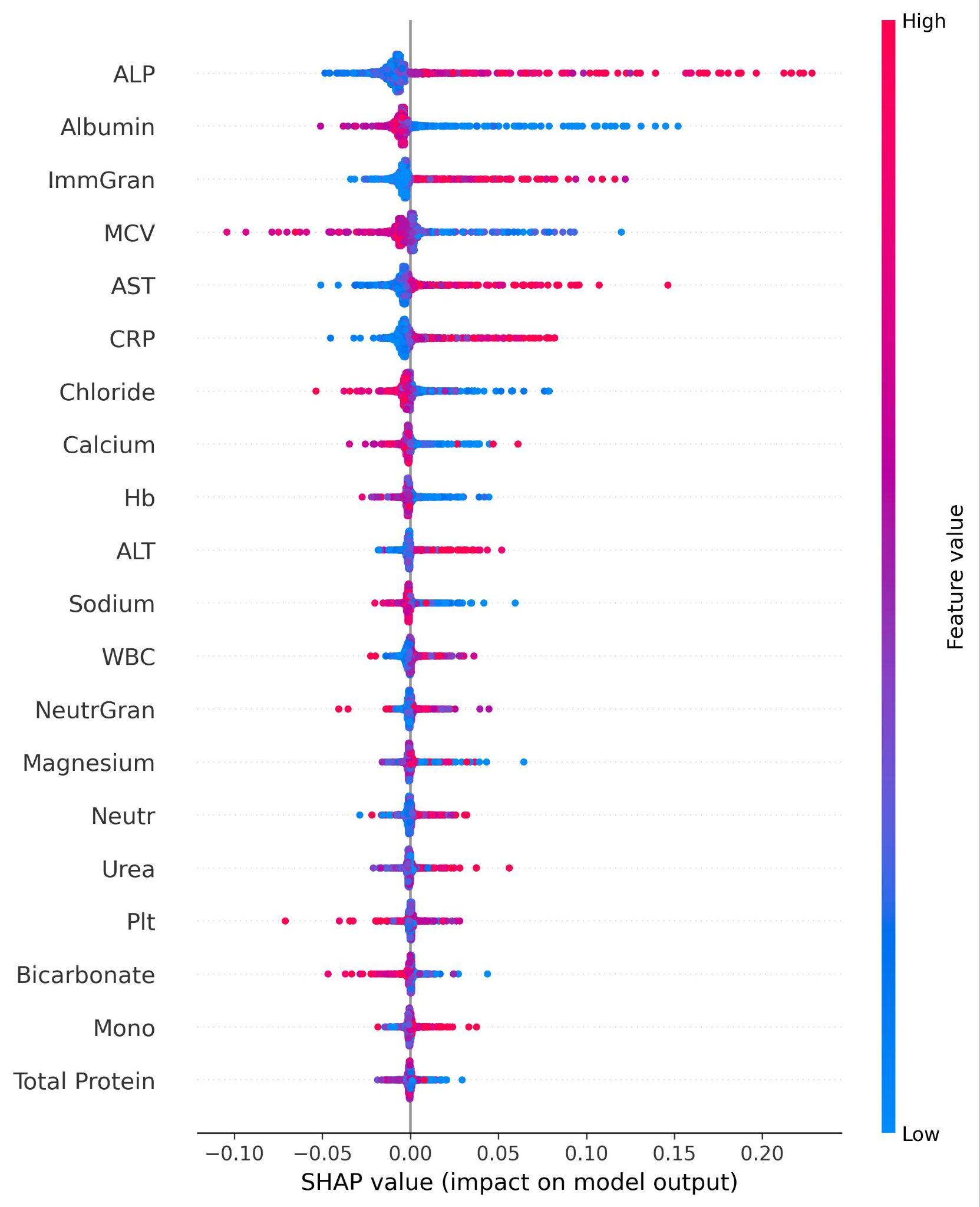


Figure 4: SHAP summary plot of 1-month pfs prediction using combined routine blood markers and tumour markers


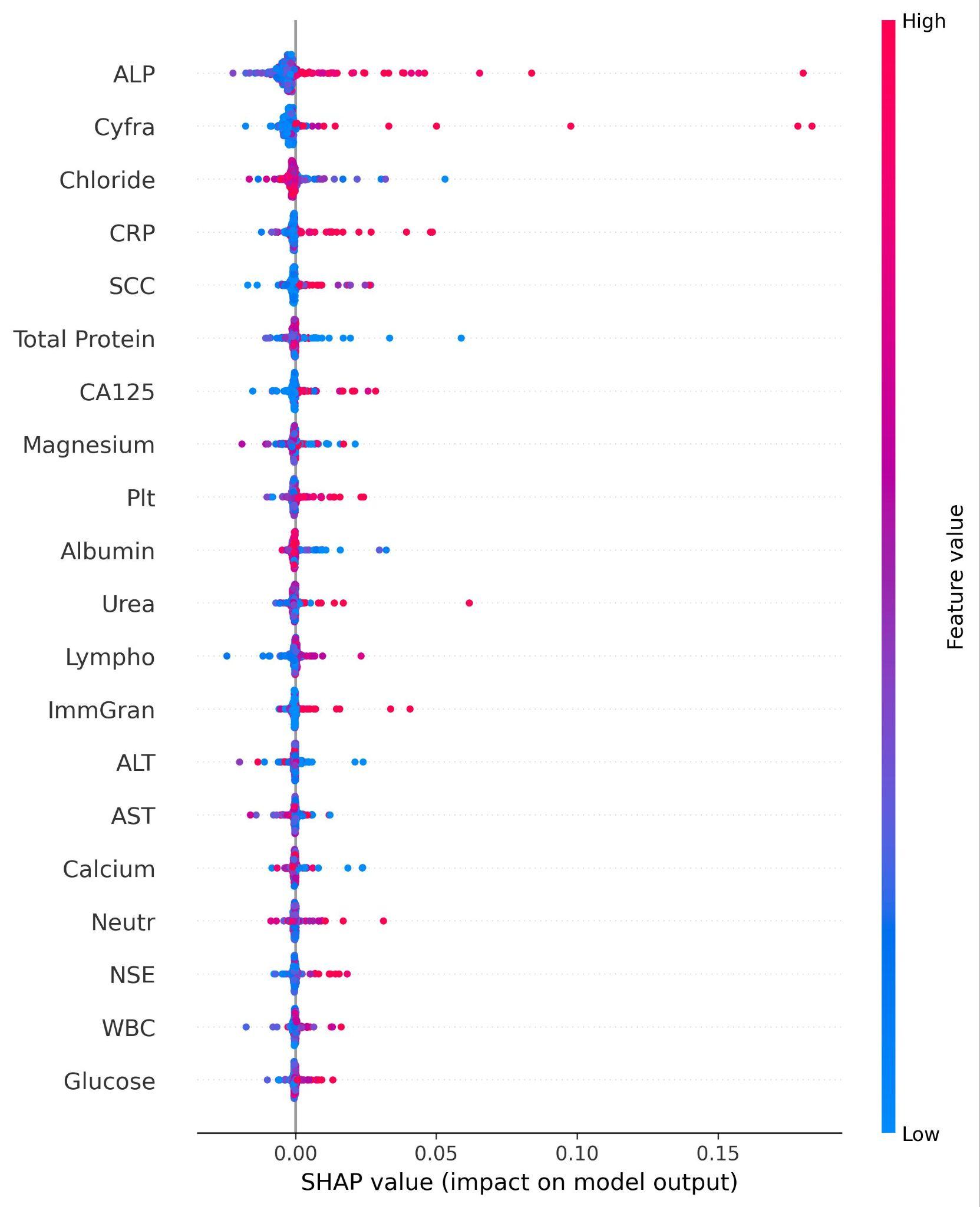


Figure 5: SHAP summary plot of 3-month pfs prediction using routine blood markers


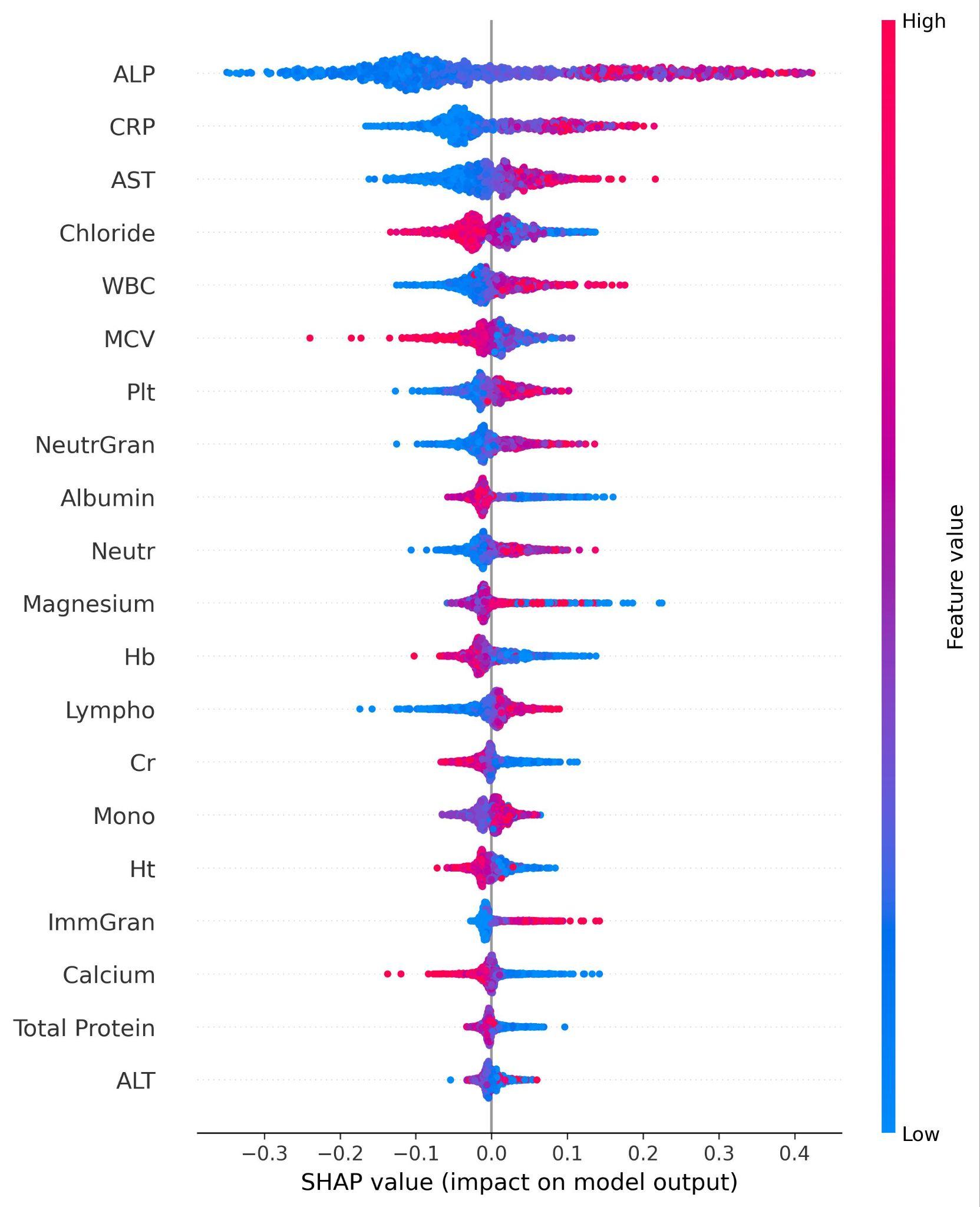


Figure 6: SHAP summary plot of 3-month pfs prediction using combined routine blood markers and tumour markers


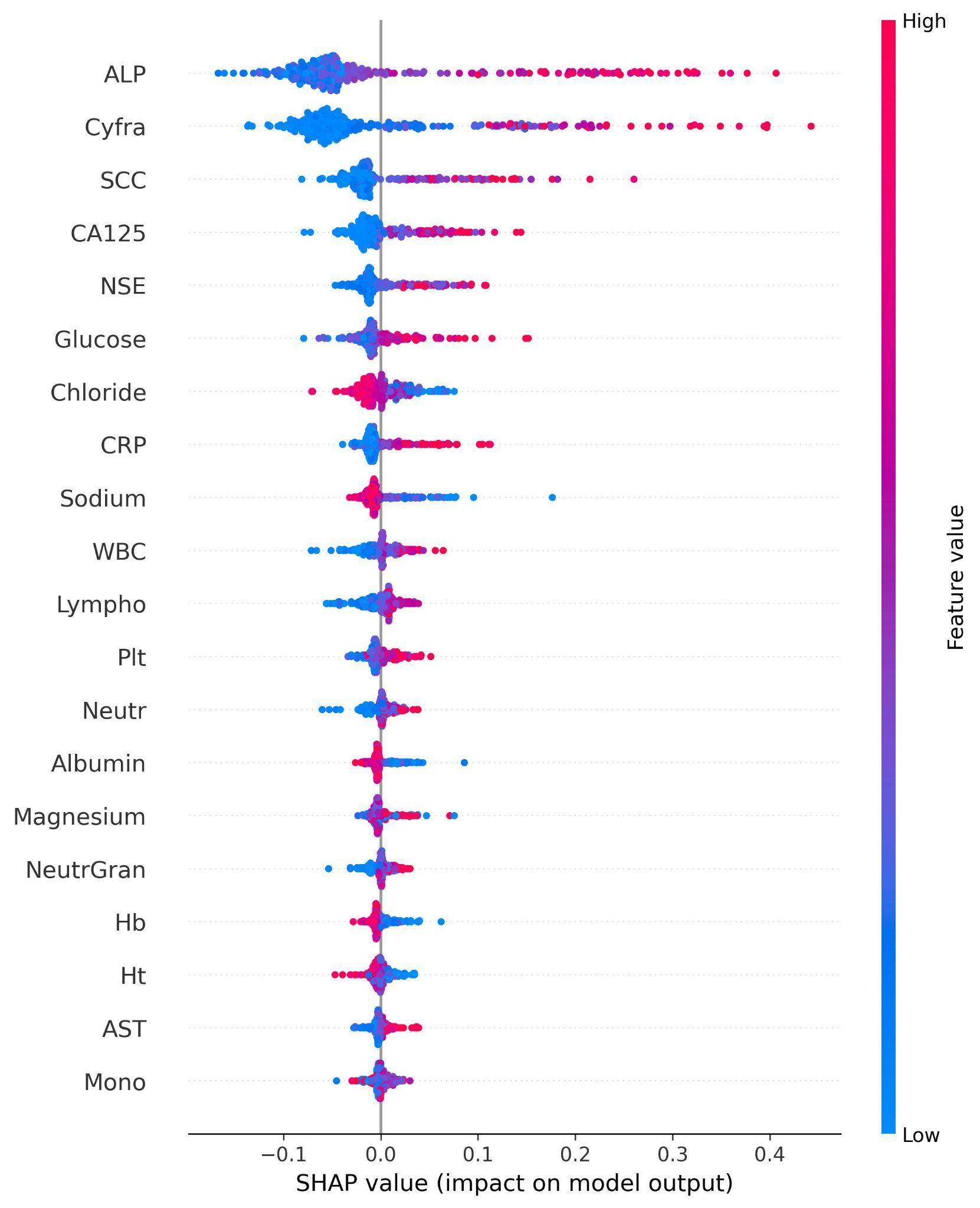


Figure 7: SHAP summary plot of 6-month pfs prediction using routine blood markers


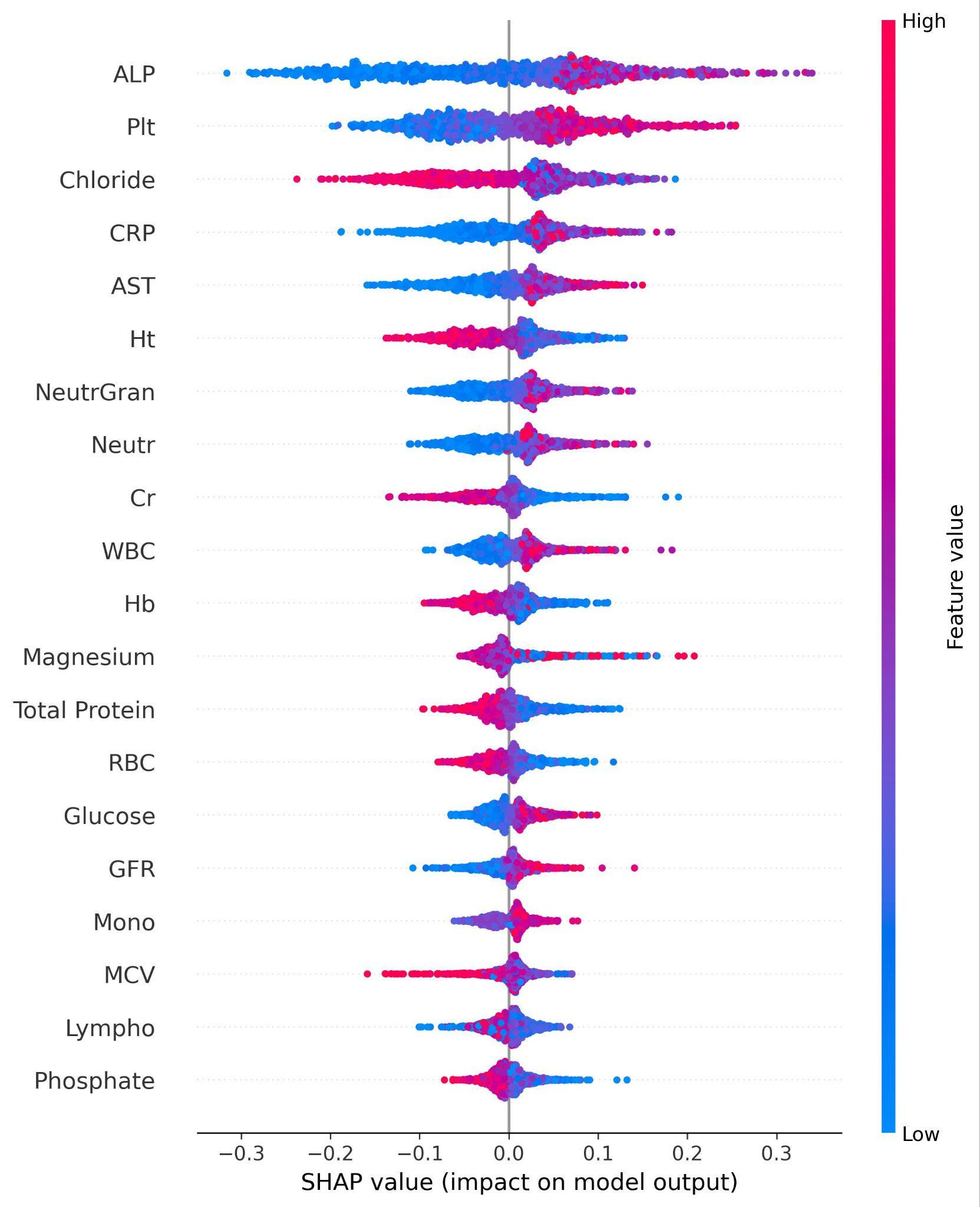


Figure 8: SHAP summary plot of 6-month pfs prediction using combined routine blood markers and tumour markers


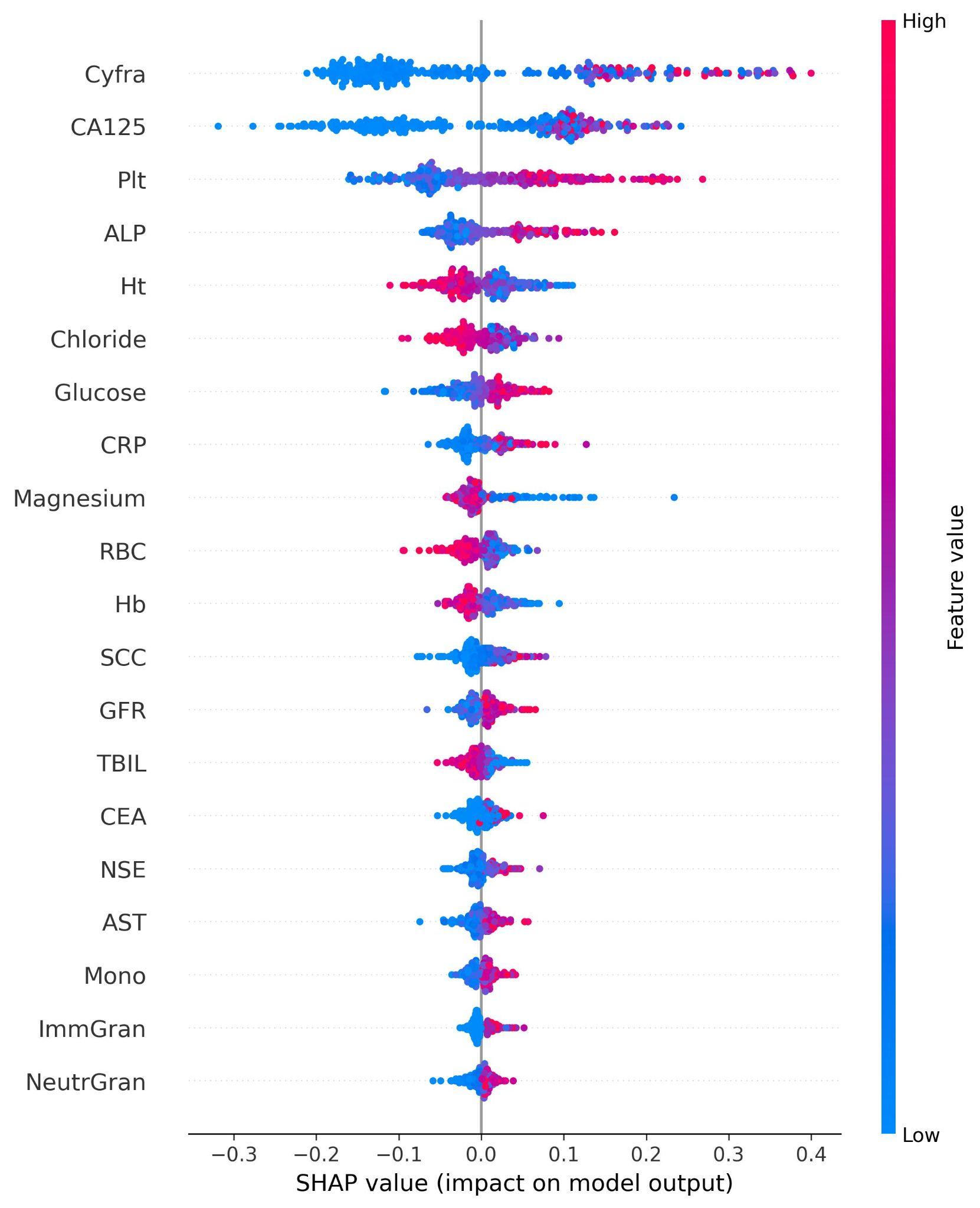


Figure 9: SHAP summary plot of 9-month pfs prediction using routine blood markers


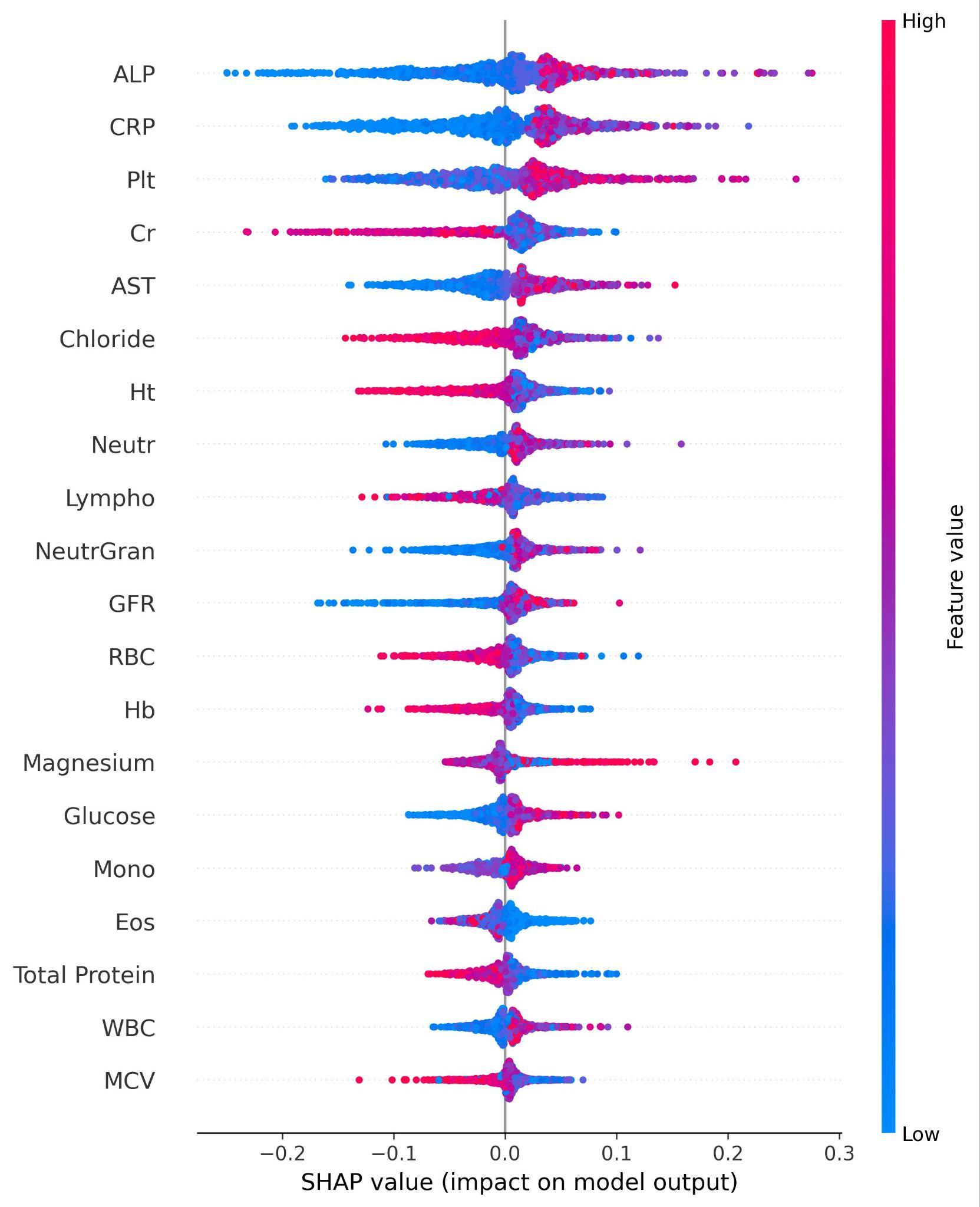


Figure 10: SHAP summary plot of 9-month pfs prediction using combined routine blood markers and tumour markers


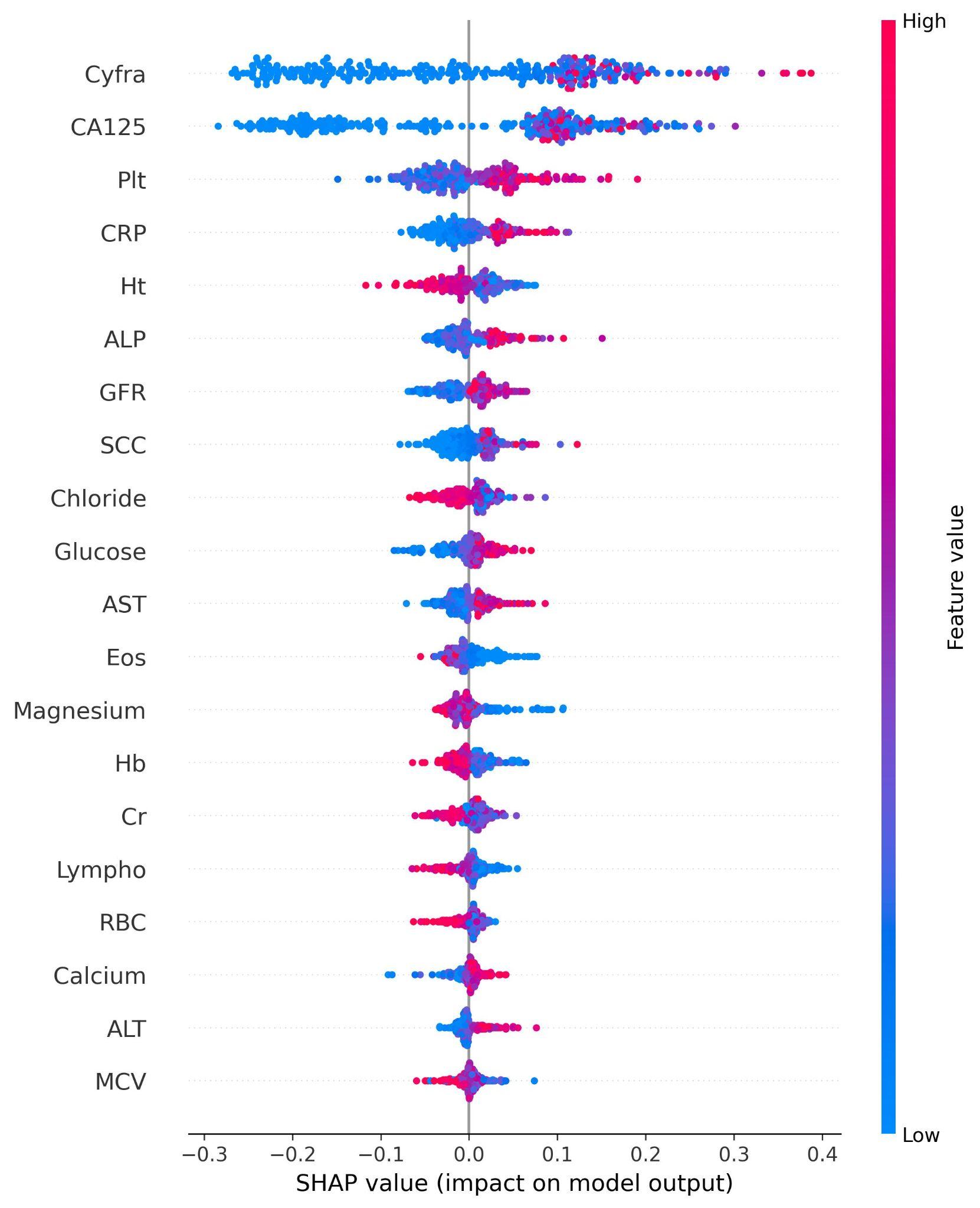


Figure 11: SHAP summary plot of 12-month pfs prediction using routine blood markers


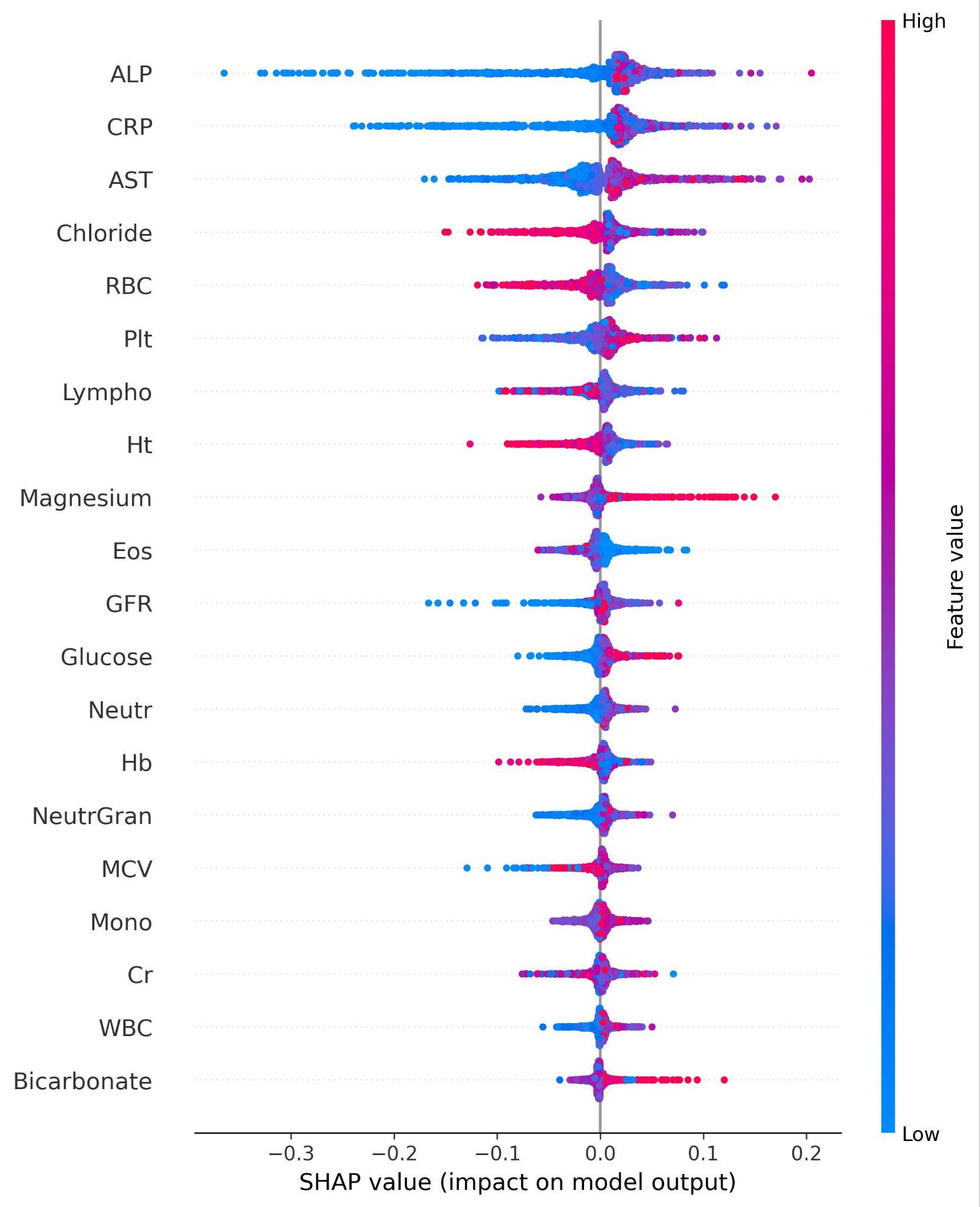


Figure 12: SHAP summary plot of 12-month pfs prediction using combined routine blood markers and tumour markers


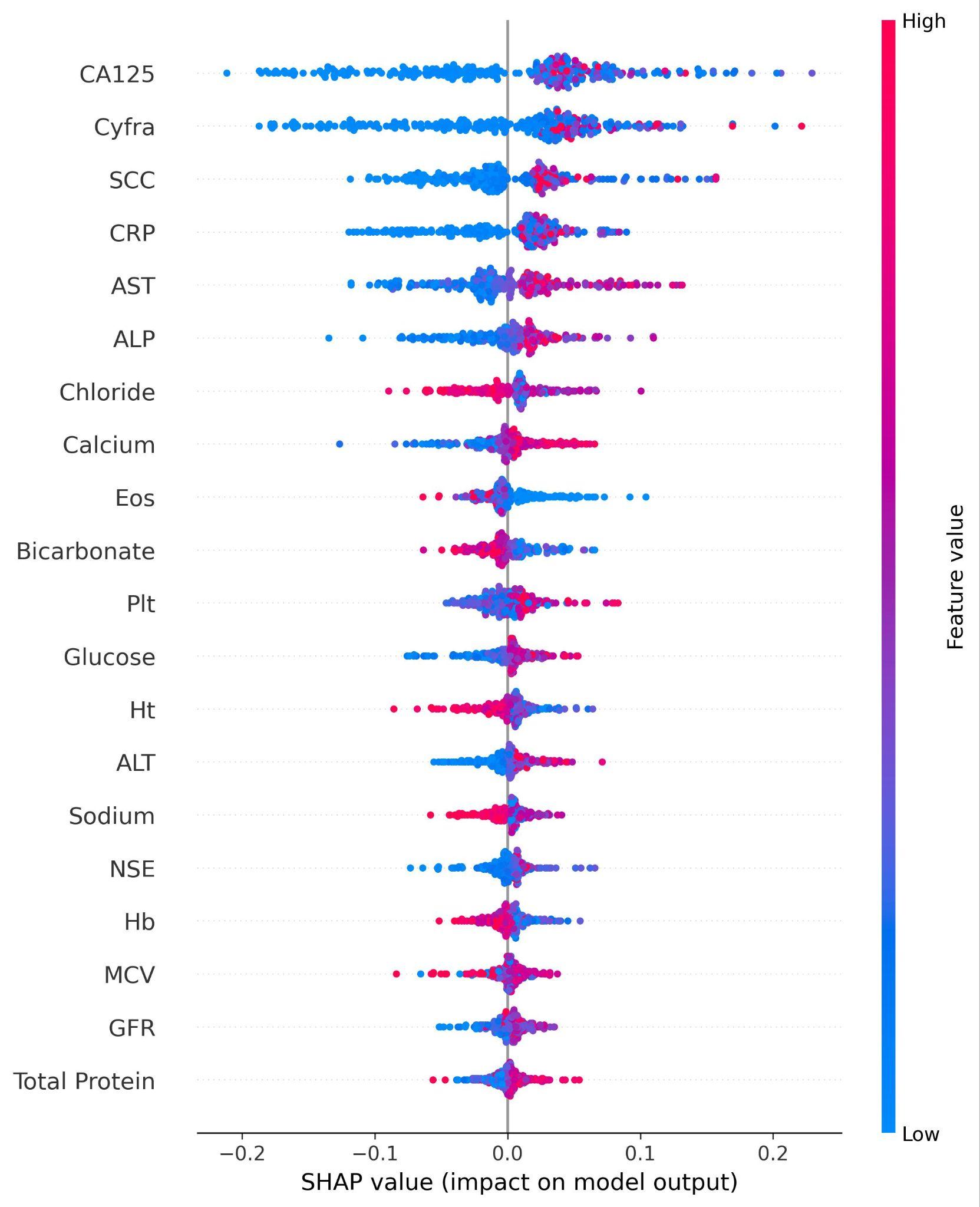

Supplement: Supplementary file 1 — Supplementary file1 (DOCX 2452 KB) [file 432_2024_5814_MOESM1_ESM.docx]
